# Allogeneic stem cell transplantation for X-linked agammaglobulinemia using reduced intensity conditioning as a model of the reconstitution of humoral immunity

**DOI:** 10.1186/s13045-016-0240-y

**Published:** 2016-02-13

**Authors:** Kazuhiro Ikegame, Kohsuke Imai, Motoi Yamashita, Akihiro Hoshino, Hirokazu Kanegane, Tomohiro Morio, Katsuji Kaida, Takayuki Inoue, Toshihiro Soma, Hiroya Tamaki, Masaya Okada, Hiroyasu Ogawa

**Affiliations:** Division of Hematology, Department of Internal Medicine, Hyogo College of Medicine, 1-1 Mukogawa-cho, Nishinomiya City, Hyogo 663-8501 Japan; Department of Community Pediatrics, Perinatal and Maternal Medicine, Graduate School of Medical and Dental Sciences, Tokyo Medical and Dental University, Tokyo, Japan; Department of Pediatrics and Developmental Biology, Graduate School of Medical and Dental Sciences, Tokyo Medical and Dental University, Tokyo, Japan

**Keywords:** X-linked agammaglobulinemia, Allogeneic transplantation, Reduced intensity conditioning, Immune recovery

## Abstract

**Background:**

We herein report the first case of X-linked agammaglobulinemia (XLA) that underwent allogeneic stem cell transplantation using reduced intensity conditioning (RIC). We chronologically observed the reconstitution of humoral immunity in this case.

**Case presentation:**

The patient was a 28-year-old Japanese male with XLA who previously had life-threatening infectious episodes and was referred for the possible indication of allogeneic stem cell transplantation. After a thorough discussion within specialists from different backgrounds, we decided to perform allogeneic peripheral stem cell transplantation from his HLA-identical elder brother. Due to the non-malignant nature of XLA, we selected RIC consisting of fludarabine, cyclophosphamide, anti-thymocyte globulin, and 3 Gy of total body irradiation. Neutrophil engraftment was achieved on day 11 with complete donor chimerism. No major complications, except for stage 1 skin graft-versus-host disease, were observed. The patient was discharged on day 75 and has been followed as an outpatient without any infectious episodes for more than 500 days.

**Conclusions:**

Regarding immune reconstitution, CD19^+^ cells, IgA, and IgM, which were undetectable before allogeneic stem cell transplantation (allo-SCT), started to increase in number 10 days after allo-SCT and continued to increase for more than 1 year. Anti-B antibodies appeared as early as day 10. Total IgG levels decreased after the discontinuation of IgG replacement and spontaneously recovered after day 350. However, most anti-viral IgG titers, except EB virus-virus capsid antigen IgG, disappeared after the discontinuation of IgG replacement. A seasonal vaccination to influenza was performed on day 148, with neither anti-influenza type A nor type B being positive after the vaccination. The transient transfer of allergic immunity to orchard grass was observed. Similar Bruton’s tyrosine kinase (*BTK*) expression levels in monocytes and B-cells were observed between the patient and healthy control. B-cells in the peripheral blood (PB) of the patient on day 279 showed sufficient proliferation after a CD40L and IL-21 or CD40L and CpG stimulation. Effective immunoglobulin production and class switching were also observed after a CD40L and IL-21 or CpG stimulation. Signal joint kappa-deleting recombination excision circles (sjKRECs) became positive 16 days post-SCT, increased to 6300 copies/μg DNA at 42 days, and were maintained at a high level thereafter. The recovery of T-cell receptor excision circles (TRECs) was slow, but became detectable 1 year post-hematopoietic stem cell transplantation (HSCT).

## Background

X-linked agammaglobulinemia (XLA) is a congenital immunodeficiency caused by mutations in Bruton’s tyrosine kinase (*BTK*) [1, 2]. Dysfunctions in *BTK* have been shown to impair B lymphocyte maturation and immunoglobulin production, resulting in hypogammaglobulinemia. Since cellular immunity is spared, most XLA patients are treated with the regular replacement of immunoglobulin G (IgG) products throughout their lifetime [3]. However, some patients develop significant infectious complications and their life expectancy is shortened despite standard therapies [4, 5]. Moreover, the cumulative cost of IgG replacement is very high [6].

Although allogeneic stem cell transplantation (allo-SCT) is theoretically a curative option for XLA, the potential risks accompanying allo-SCT have been a barrier to it becoming a standard therapy for XLA. Howard et al. reported the first case series of allo-SCT for XLA. They did not use a preconditioning regimen based on findings obtained in XLA model mice, in which no stable donor engraftment was achieved, resulting in no harm, but no benefit [6]. Allo-SCT was recently performed on a patient with XLA coincidentally complicated with acute myeloid leukemia (AML), in which a myeloablative conditioning regimen was used because it is the standard treatment for AML [7]. Since XLA itself is a non-malignant disorder, reduced intensity conditioning (RIC) may be suitable for cases of allo-SCT for XLA in order to minimize transplantation-associated toxicity [8]. We herein present a successful case of allo-SCT for XLA using RIC, in which we chronologically observed the clinical course of the reconstitution of humoral immunity.

### Methods

#### Flow cytometry

Intracellular *BTK* staining was performed as previously described [9]. Briefly, peripheral blood mononuclear cells (PBMCs) were labeled with phycoerythrin-conjugated anti-CD14 (Dako) or CD19 (Beckman Coulter). Cells were fixed, permeabilized, stained with 2 μg/mL of anti-*BTK* (clone 10E10, OriGene Technologies, Inc.) or isotype monoclonal antibodies (BD Biosciences), and subsequently stained with 1:2000 dilution of fluorescein isothiocyanate-conjugated anti-mouse IgG2a (Southern Biotechnology Associates, Inc.). Stained cells were analyzed using BD LSRFortessa (BD Biosciences), and data were processed using FlowJo software (Tree Star Inc.).

#### Proliferation assay

PBMCs were labeled with CFSE (3 μM; eBioscience) at room temperature for 5 min and stimulated for 4 days with a CD40 ligand (CD40L, 1 μg/mL; Miltenyi Biotec) and CpG (1 μg/mL; InvivoGen) or CD40L (1 μg/mL) and IL-21 (50 ng/mL; Miltenyi Biotec). Cells were then stained for CD19 and analyzed using flow cytometry.

#### In vitro immunoglobulin production assay

An in vitro immunoglobulin production assay was performed as previously described [10]. Briefly, PBMCs were stimulated with CpG (1 μg/mL) or the CD40 ligand (1 μg/mL) and IL-21 (100 ng/mL) and then cultured for 12 days. Immunoglobulin levels in the culture supernatants were measured using ELISA. Pooled human serum with known concentrations of IgG, IgA, and IgM was used as the standard. The sensitivities of the assays used were as follows: IgG and IgA, 5 ng/mL, and IgM, 10 ng/mL.

#### TREC analysis

The levels of T-cell receptor excision circles (TRECs), signal joint kappa-deleting recombination excision circles (sjKRECs), and coding joint KRECs (cjKRECs) were measured by real-time PCR as described previously [11]. RNase P was used as an internal control. Primer and probe sequences were listed previously [12]. The minimum detectable limit was 10 copies/μg DNA.

## Case presentation

The patient was a 28-year-old Japanese male who had been diagnosed with XLA soon after his birth. He had been receiving IgG replacement therapy since he was an infant. Regarding his previous infectious history, he had chronic sinusitis, which twice required surgery, episodes of chronic upper respiratory infections, cellulitis progressing to osteomyelitis, tympanomastoiditis, and meningitis resulting in cerebral hemorrhage. Each episode was refractory to antibiotic therapy and potentially fatal. He was referred to our hospital for the possible indication of allo-SCT. The diagnosis of XLA was confirmed by the direct sequencing of *BTK* gene mutations [13]. He had an HLA-identical elder healthy brother. After a thorough discussion with pediatricians specializing in hematology, immunodeficiency, and transplantation, we decided to perform allo-SCT from his elder brother. The risks associated with allo-SCT were explained to the patient and his family including the donor. Written informed consent was obtained from the patient and his family. HLA of the patient and his elder brother, the donor, was HLA-A*02:01/24:02 B*40:01/51:01 C (not tested) DRB1*11:01/14:03, and the blood type of both was A+.

We used a RIC regimen consisting of fludarabine (FLU) at 30 mg/m^2^/day for 6 days (days −10 to −5), cyclophosphamide (CY) at 50 mg/kg/day for 2 days (days −4 and −3), 3 Gy of total body irradiation (TBI), and rabbit anti-thymocyte globulin (ATG, Thymoglobulin, Sanofi) at 1.25 mg/kg/day for 2 days (days −2 and −1). In order to prevent anaphylaxis by ATG, methylprednisolone (mPSL) was administered at 2 mg/kg on days −2 and −1. Prophylaxis for graft-versus-host disease (GVHD) consisted of the continuous infusion of cyclosporine (CsA) starting at a dose of 0.3 mg/kg/day from day −2 and oral mycophenolate mofetil (MMF) starting at a dose of 30 mg/kg/day from day 2. The target blood concentration of CsA was set at 500–550 ng/mL up to day 20, 350–500 ng/mL on days 20–35, and thereafter switched to oral CsA. Peripheral blood stem cell (PBSC) mobilization using granulocyte colony-stimulating factor (G-CSF) was performed as described previously [14]. Briefly, leukapheresis was performed twice on days 4 and 5 of the G-CSF administration. PBSCs were freshly transplanted on days 0 and +1. The total dose of PBSCs contained 6.66 × 10^8^ nucleated cells/kg and 4.77 × 10^6^ CD34^+^ cells/kg. After transplantation, the patient received G-CSF from day 5 to the day of neutrophil engraftment.

**Fig. 1:**
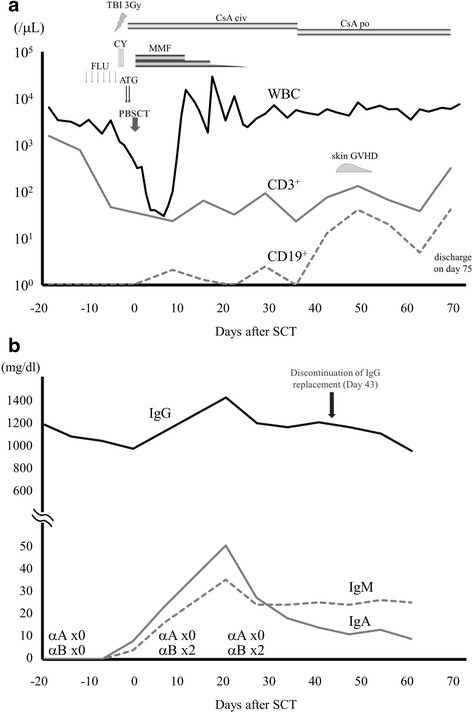
Clinical course associated with the recovery of T and B lymphocytes (**a**) and serum levels of immunoglobulins (**b**) in the admitted period. **a** The clinical course was illustrated. Reduced intensity conditioning (RIC) consisted of fludarabine (*FLU*), cyclophosphamide (*CY*), and 3 Gy of total body irradiation (*TBI*). Anti-thymocyte globulin (*ATG*, Thymoglobulin, Sanofi) was added for the purpose of prophylaxis for rejection and graft-versus-host disease (*GVHD*). GVHD prophylaxis consisted of cyclosporine (*CsA*) and mycophenolate mofetil (*MMF*). The numbers of CD3^+^ (*solid gray*) and CD19^+^ (*dotted gray*) lymphocytes in the peripheral blood (PB) were followed in the admitted period. **b** Serum immunoglobulin levels in the admitted period were plotted. Whereas IgA (*solid gray*) and IgM (*dotted gray*) were not detected before allo-SCT, the titers of both rapidly increased within 1 week of allo-SCT and peaked on day 20. Anti-B antibodies (the blood type of the recipient and donor was A+) were also detected as early as day 10. IgG (*solid black*) levels decreased after the discontinuation of IgG replacement therapy and were maintained at more than 500 mg/dL

The clinical course of allo-SCT was shown in Fig. 1. Hematopoietic engraftment was rapidly achieved, with an absolute neutrophil count of >0.5 × 10^9^/L on day 11 and platelet count of >50 × 10^9^/L on day 25. The donor chimeras of CD3^+^ and neutrophil fractions in PB were 100 and 68.2 %, respectively, on day 3 using an informative short tandem repeat (STR)-PCR technique, and 100 % donor chimerism was confirmed in the CD3^+^ and neutrophil fractions on day 10. Since there was no evidence of GVHD, MMF was discontinued on day 23. Stage 1 skin GVHD developed on day 44, which disappeared after slightly increasing the dose of CsA. Weekly IgG replacement was discontinued on day 43. He was discharged on day 75 and has been doing well without any infectious episodes until the last day of the follow-up on day 500.

### Chronological observations of immune recovery

**Fig. 2:**
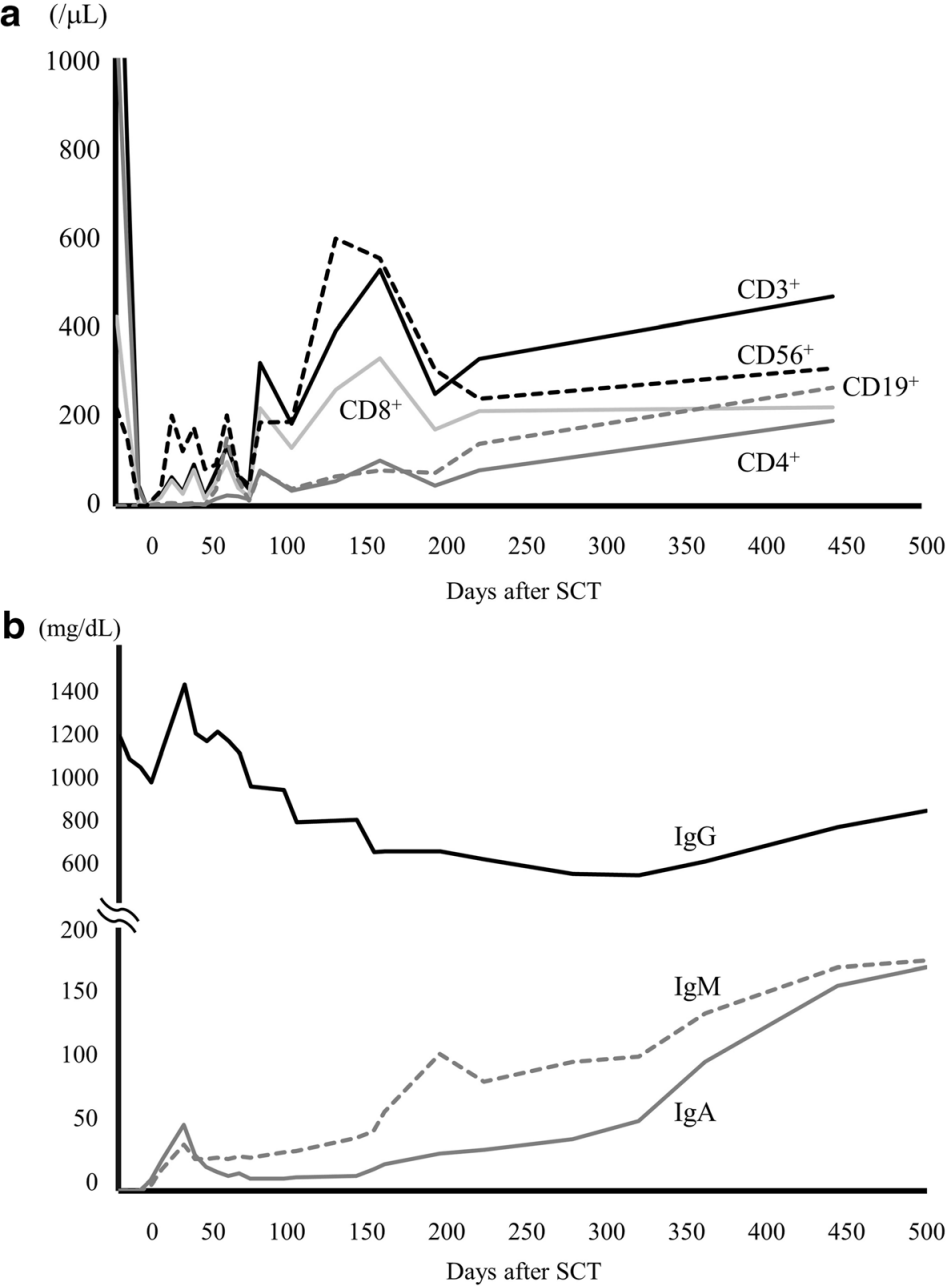
Recovery of lymphocyte fractions (CD3^+^ (*solid black*), CD4^+^ (*solid thick gray*), CD8^+^ (*solid thin gray*), CD19^+^ (*dotted gray*), and CD56^+^ (*dotted black*)) (**a**) and serum levels of immunoglobulins (**b**) in the long-term follow-up period. The total level of IgG (*solid black*) has been maintained at more than 500 mg/dL without supplementation and has gradually increased. IgA (*dotted gray*) and IgM (*solid gray*) have been gradually increasing to normal ranges, with a marked increase being observed in IgM

Lymphocyte recoveries during the admitted and long-term follow-up periods were shown in Figs. 1a and 2a, respectively. CD19^+^ cells, which were undetectable before allo-SCT, started to increase in number 10 days after allo-SCT and continued to increase for more than 1 year. On the other hand, CD3^+^ cells decreased by an order of 10^1^/μL and recovered by an order of 10^2^/μL. Regarding comparisons of CD3^+^ and CD56^+^ cells, CD56^+^ cells were dominant until day 250, with the number of CD3^+^ cells thereafter increasing to more than that of CD56^+^ cells. Regarding comparisons of CD4^+^ and CD8^+^ cells, CD8^+^ cells were always more dominant than CD4^+^ cells.

The recovery of immunoglobulins attracted our interest in this case. IgA and IgM, which were undetectable before allo-SCT, rapidly increased by day 20 (Fig. 1b). Since the blood type of the patient and donor was A+, we followed the titer of anti-B antibodies and found that it appeared as early as on day 10. IgG levels decreased after the discontinuation of IgG replacement and gradually recovered from day 350 (Fig. 2b). Regarding antibodies to several viruses, all anti-viral IgG, except for EB virus-virus capsid antigen IgG (EBV-VCA IgG), disappeared after the discontinuation of IgG replacement (Table 1). All anti-viral IgM remained negative before and after allo-SCT. A seasonal vaccination to influenza was performed on day 148, with neither anti-influenza type A nor type B being positive after the vaccination.

**Table 1:**
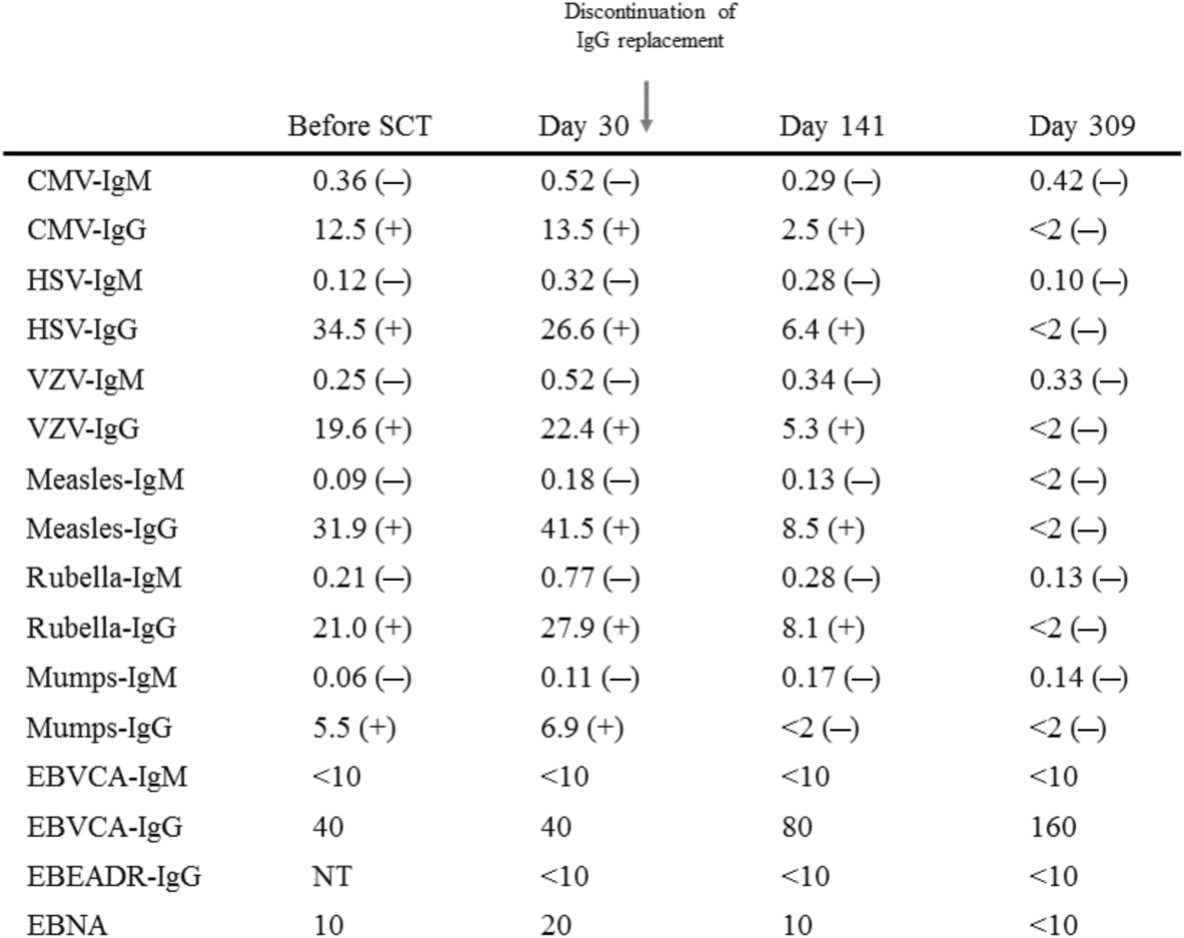
Virus-specific IgM and IgG antibodies

The titers of virus-specific antibodies were monitored before and after allo-SCT. IgG titers disappeared after the discontinuation of IgG replacement, except for EBV-VCA IgG

*NT* not tested

We then examined data related to allergies. The radioimmunosorbent test (RIST), which reflects the total amount of IgE, was undetectable on days 2 and 46 but became positive on day 393 after allo-SCT. Among the various allergens tested, only the radioallergosorbent test (RAST) to orchard grass, to which the donor had a high titer of RAST, slightly increased after allo-SCT (day 46) (Table 2). The donor has seasonal hay fever to orchard grass, whereas the patient has no symptoms of hay fever.

**Table 2:** Allergic responses

	Standard	Donor	Day −12	Day 46	Day 393
IgE-RIST	(0–173)	340	<5	<5	23.9
Orchard grass	(0–0.34)	67.7	<0.1	<0.26	<0.10
Cedar	(0–0.34)	24.4	<0.1	<0.1	<0.1
Cypress	(0–0.34)	3.34	<0.1	<0.1	<0.1
*Dermatophagoides pteronyssinus*	(0–0.34)	0.2	<0.1	<0.1	<0.1
*Dermatophagoides farinae*	(0–0.34)	0.19	<0.1	<0.1	<0.1
Cat’s dandruff	(0–0.34)	<0.1	<0.1	<0.1	<0.1
House dust 1	(0–0.34)	0.16	<0.1	<0.1	<0.1
House dust 2	(0–0.34)	0.20	<0.1	<0.1	<0.1

The radioimmunosorbent test (RIST) indicating the total amount of IgE and radioallergosorbent test (RAST) indicating allergen-specific IgE was monitored before and after allo-SCT. RIST was undetectable until day 46 and became positive on day 393. The titer of RAST specific to orchard grass, to which the donor had a high titer of RAST, slightly increased on day 46

**Fig. 3:**
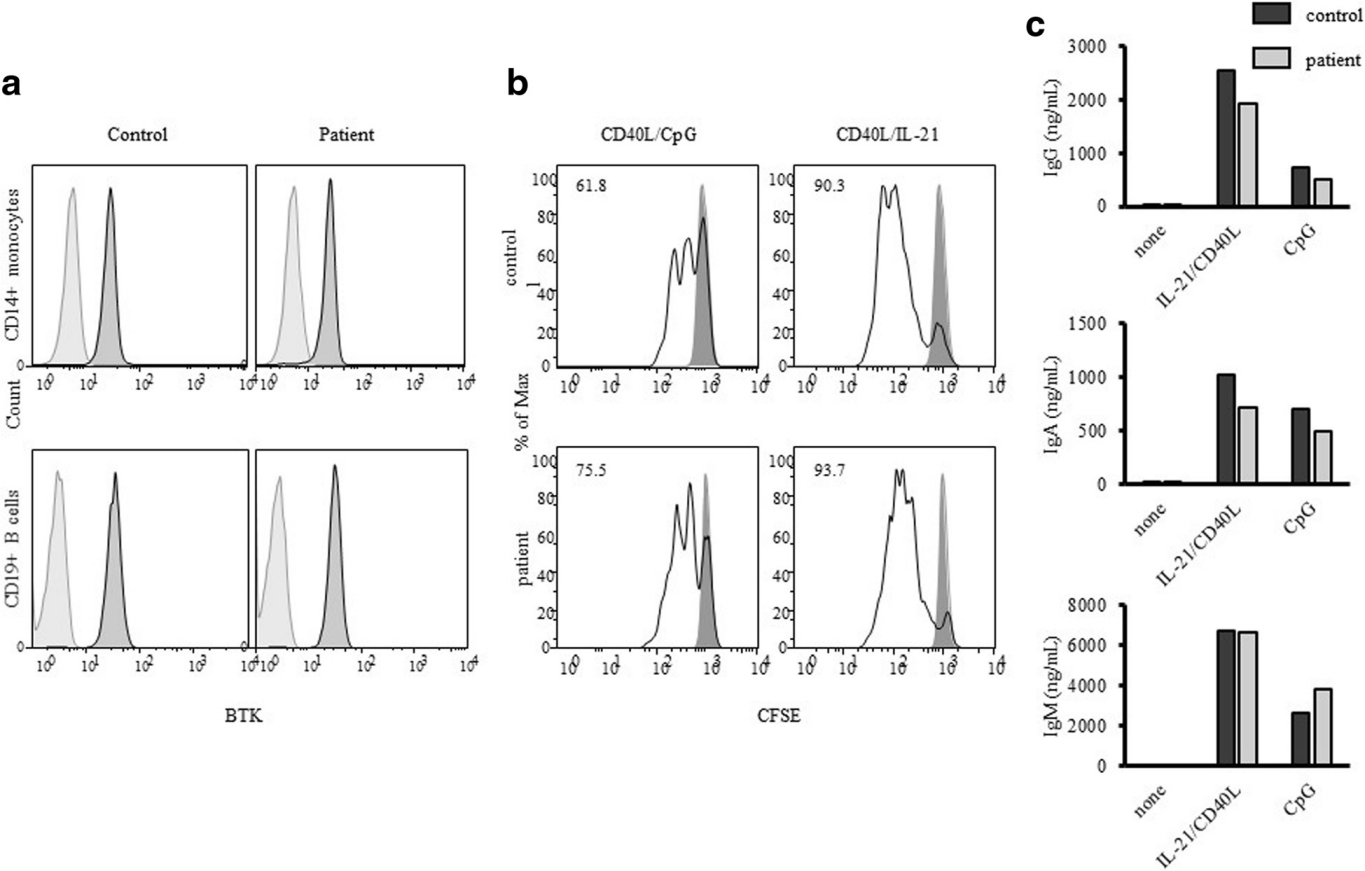
Intracellular *BTK* expression and B-cell functional responses. A blood sample collected from the patient on day 279 was used for these assays. **a** Flow cytometric analysis of *BTK* expression in monocytes and B-cells. *Gray histograms* indicate isotype control and *black histograms* indicate *BTK* expression. **b** CFSE-labeled proliferation induced by CD40L and CpG or CD40L and IL-21 in CD19^+^ B-cells. *Numbers in the plots* indicate the percentage of divided cells. **c** Immunoglobulin production induced by CD40L and IL-21 or CpG

**Fig. 4:**
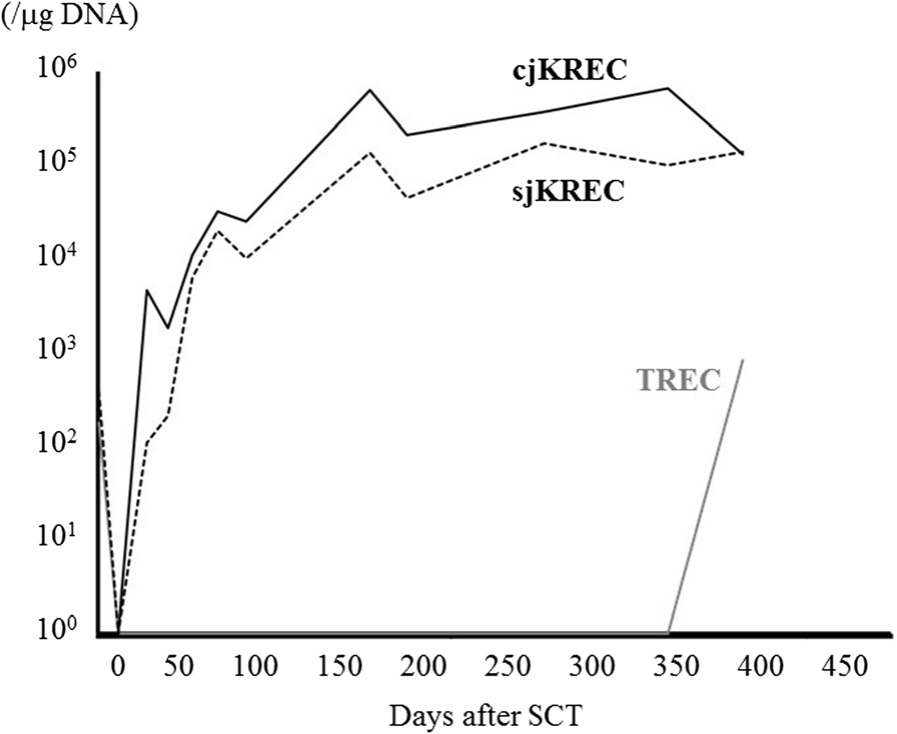
TRECs and KRECs as markers of T- and B-cell neogenesis. sjKRECs (*dotted black*), which serve as an indicator of B-cell neogenesis, became positive 16 days post-SCT, increased to 6300 copies/μg DNA at 42 days, and were maintained at a high level thereafter. cjKRECs (*solid black*), an indicator of B-cell numbers, fluctuated almost in parallel with sjKRECs. The recovery of TRECs (*solid gray*) was slow, but became detectable 1 year post-HSCT

We also performed functional tests. *BTK* protein expression levels and B-cell functional responses were evaluated on day 279 (Fig. 3a). Similar *BTK* expression levels in monocytes and B-cells were observed between the patient and healthy control as expected. In order to investigate B-cell functional responses, an in vitro proliferation assay (Fig. 3b) and immunoglobulin production assay (Fig. 3c) were performed. B-cells from the patient showed sufficient proliferation after a CD40L and IL-21 or CD40L and CpG stimulation. Effective immunoglobulin production and class switching were also observed after a CD40L and IL-21 or CpG stimulation. In order to assess T-cell and B-cell neogenesis, we monitored data for TRECs, cjKRECs, and sjKRECs (Fig. 4). sjKRECs, which serve as an indicator of B-cell neogenesis, became positive 16 days post-SCT, increased to 6300 copies/μg DNA at 42 days, and were maintained at a high level thereafter. B-cell recovery was superior to the previously reported time course of sjKRECs post-hematopoietic stem cell transplantation (HSCT) [12]. The recovery of TRECs was slow, but became detectable 1 year post-HSCT. The patient achieved immune reconstitution with a normal immune cell configuration judging from a flow cytometric analysis of PBMCs (data not shown).

### Discussion

XLA is a congenital humoral immunodeficiency caused by *BTK* gene mutations. A standard treatment for XLA is regular IgG replacement, through which a long life expectancy may be achieved [3]. However, some patients develop chronic infectious episodes despite IgG replacement and have a shorter life expectancy [4, 5]. Theoretically, allo-SCT is a curative option for XLA; however, the risk of treatment-related mortality has been a barrier to allo-SCT being performed for XLA. The first case series of allo-SCT for XLA was reported by Howard et al., in which six XLA patients received transplants from their HLA-matched siblings without a preconditioning regimen, based on findings obtained in XLA model mice, X-linked immunodeficiency *(xid)* [6]. In contrast to *xid*, stable donor engraftment was not achieved in human cases, and resulted in no harm, but to no benefit. Abu-Arja et al. recently reported successful allo-SCT in a patient with XLA and AML, in which they used a myeloablative conditioning regimen because it is the standard treatment for AML [7]. They suggested the use of a RIC regimen in the case of allo-SCT for XLA. In the present study, we described a case of allo-SCT for XLA using RIC (Table 3).

**Table 3:** Reports of allogeneic stem cell transplantation (allo-SCT) for XLA

	Case number	Preconditioning	GVHD prophylaxis	Neutrophil engraftment	Increase of CD19^+^ and Ig	Reference
Howard et al.	*N* = 1	(−)	(−)	No	No	[6]
	*N* = 3	(−)	CsA/MMF	No	No	[6]
Abu-Arja et al.	*N* = 1	ETP/CY/12Gy	TAC/MTX	Yes	Yes	[7]
Ikegame et al.	*N* = 1	FLU/CY/ATG/3Gy	CsA/MMF	Yes	Yes	This report

There are two English reports of allo-SCT for XLA. Howard et al. present six cases based on their experience, three of which underwent SCT without preconditioning or GVHD prophylaxis, while the other three underwent GVHD prophylaxis consisting of cyclosporine and mycophenolate mofetil (CsA/MMF). No patients achieved donor engraftment or increases in CD19^+^ cell numbers or immunoglobulin (Ig) levels [6]. Abu-Arja et al. reported successful allo-SCT in a patient with XLA and AML, in which they used a myeloablative conditioning regimen consisting of etoposide 40 mg/kg for 1 day, cyclophosphamide (CY) 60 mg/kg/day, and 12 Gy of total body irradiation (ETP/CY/12Gy), and GVHD prophylaxis consisting of tacrolimus and methotrexate (TAC/MTX). The patient achieved engraftment with donor chimerism and had normal CD19^+^ cell numbers and Ig levels. In our case, the preconditioning regimen used comprised fludarabine, CY, rabbit anti-thymocyte globulin, and 3 Gy of TBI (FLU/CY/ATG/3Gy), and GVHD prophylaxis was CsA and MMF (CsA/MMF). As described in the text, the patient achieved engraftment with donor chimerism, with a rapid increase in CD19^+^ cell numbers and the production of Ig. To the best of our knowledge, this is the first English case report of successful allo-SCT for XLA

Due to the benign nature of XLA, a discussion on preconditioning is critical. Baron et al. previously reported the findings of a phase II multicenter randomized study comparing non-myeloablative allo-SCT with either FLU plus 2 Gy of TBI (FLU-TBI) or 8 Gy of total lymphoid irradiation plus ATG (TLI-ATG) [15]. The TLI-ATG regimen reduced the 2-year cumulative incidence of moderate/severe chronic GVHD to 17.8 % and increased the 4-year cumulative incidence of relapse/progression to 50 %. These findings indicate that the suppression of chronic GVHD by ATG ameliorates graft-versus-leukemia (GVL) effects and further relapses, thereby offsetting the benefit of low GVHD by a high relapse rate and resulting in the same overall survival rate in malignant diseases. Since XLA is a benign disease and has no GVL effects, the use of ATG may be beneficial to allo-SCT for XLA. As an example of a regimen for adult non-malignancies, Bacigalupo et al. employed FLU/CY/ATG with or without low-dose TBI to treat acquired severe aplastic anemia [16]. They demonstrated that the rejection rate was high in older patients without TBI, and some centers in Europe have introduced low-dose TBI. Although the regimen of busulfan, FLU, and ATG may be another option, a high graft failure rate (21 %) was reported for children with non-malignant disorders and chronic myelogenous leukemia [17]. Arai et al. previously demonstrated the significance of high-dose cytarabine added to CY/TBI for bone marrow transplantation (BMT) and peripheral blood stem cell transplantation (PBSCT) for myeloid malignancy [18]. In contrast to the findings obtained for cord blood stem cell transplantation, no additional benefit was observed in BMT or PBSCT, which did not justify the addition of cytarabine to the regimen in this case. Regarding GVHD prophylaxis, although the combination of CsA plus methotrexate (CsA/MTX) is the current standard for SCT from HLA-identical sibling donors, MMF in combination with a calcineurin inhibitor, tacrolimus, or CsA has recently been highlighted for its potential in achieving rapid hematopoietic recovery and mild mucosal toxicity [19]. A head-to-head comparison of MMF in combination with CsA (CsA/MMF) and CsA/MTX was performed by Piñana et al., in which they showed that CsA/MMF suppressed acute and chronic GVHD to the same extent as CsA/MTX and also reduced the incidence of mucositis more than CsA/MTX after RIC PBSCT [20]. Lai et al. more recently reported the beneficial outcomes of a combination of CsA, MTX, and MMF (CsA/MTX/MMF) [21]. It has not yet been established whether prolonged neutrophilia and strong immunosuppression by CsA/MTX/MMF counterbalances SCT for benign immunodeficiency disorders. Based on these findings, we prefer to use a RIC regimen consisting of FLU, CY, ATG, and 3 Gy of TBI followed by prophylaxis for GVHD consisting of CsA and MMF for adult patients with non-malignant disorders, such as aplastic anemia or paroxysmal nocturnal hemoglobulinemia, which may lead to stable donor chimerism without severe regimen-related toxicity [22]. We applied this regimen to our XLA case in order to minimize toxicity and achieve the permanent reconstitution of the immune system.

In spite of the advances in SCT techniques described above, the indication of allo-SCT for immunodeficiency disorders needs to be carefully considered in each case. Wehr et al. summarized multicenter experiences in allo-SCT for common variable immunodeficiency (CVID) [23]. The reasons for selecting allo-SCT included lymphoma (24 %), severe infections (12 %), and complex immunological dysfunctions such as cytopenia and inflammatory organ involvement (60 %). Although allo-SCT in patients with CVID was beneficial in most surviving patients, high mortality was associated with GVHD and infectious complications. The indication of allo-SCT for XLA is also difficult to define due to conflicting findings on the mortality rate of XLA patients [4]. Although most XLA patients may experience a nearly normal life [5], Abolhassani et al. reported that 26.8 % of patients died during a follow-up period of 20 years [24]; the hospitalization rate for patients was significantly higher than alive patients. The cause of death among the 11 patients was mainly end-stage of chronic lung disease in addition to one case of meningitis. According to a national survey conducted in Japan, the 5-year survival rate of allo-SCT for aplastic anemia from related donors was more than 80 %. Due to advances in the SCT technique, repetitive episodes of hospitalization and/or central nervous system lesions may facilitate allo-SCT as a treatment option for XLA.

Before discussing immune reconstitution in this case, the possible impact of ATG needs to be considered from qualitative and quantitative aspects. Two kinds of rabbit ATGs are commonly used in SCT conditioning: anti-Jurkat T-cell line globulin (thymoglobulin) and anti-thymocyte globulin Fresenius (ATG-F). ATG-F was previously reported to affect the reconstitution of not only T-cells but also B-cells [25]. Since we used thymoglobulin in this case, its impact on B-cells was considered to be minimal. Admiraal et al. found a relationship between the dose of ATG and immune reconstitution in pediatric SCT. The commonly used dose regimen of thymoglobulin in SCT is 10 mg/kg worldwide. They found that the area under the curve of ATG increased, the achievement of successful immune recognition (defined as CD4^+^ T-cells >0.05 × 10^9^ cells per liter in two consecutive measurements within 100 days) decreased, and successful immune reconstitution by day 100 was associated with an increase in overall survival caused by reduced non-relapse mortality and relapse-related mortality [26]. Fu et al. showed that the incidence of late-onset hemorrhagic cystitis after haploidentical SCT was higher in high-dose ATG (thymoglobulin 10 mg/kg) than in low-dose ATG (thymoglobulin 6 mg/kg) [27]. Since we previously demonstrated that even a low dose of ATG, thymoglobulin 2.5 mg/kg, was sufficient to control GVHD at an acceptable level in the Japanese population based on our experience of more than 500 cases of haploidentical SCT [28], we used thymoglobulin 2.5 mg/kg in this case and considered the effects of ATG on T-cell recovery to have also been minimized.

In our case, CD19^+^ cells in PB rapidly increased in number after allo-SCT, and this was accompanied by IgA and IgM production. Moreover, anti-B antibody production, as an example of specific antibody production, was observed as early as on day 10. Although the total amount of IgG in PB spontaneously recovered, the titer of virus-specific antibodies, except for EBV-VCA IgG, disappeared after the discontinuation of IgG replacement. A vaccination for influenza virus was unsuccessful in the first season after allo-SCT. Thus, pathogen-specific immune responses had not fully recovered 1 year after allo-SCT. This may be explained by T-cell reconstitution not being thoroughly achieved, as shown by the TREC analysis (Fig. 4). Nevertheless, the patient has been doing well without any infectious episodes, which had previously occurred and were life-threatening. Another interesting result was the possible transfer of immune or allergic responses from the donor. In this setting, the donor had a high titer of RIST and RAST to some allergens. The recipient exhibited a slight increase in RAST to orchard grass on day 46, implicating the transient transfer of allergic immunity.

## Conclusions

Although we had to carefully observe the clinical course because of the short follow-up period, allo-SCT with RIC induced rapid and stable engraftment without any severe complications. IgA and IgM started to increase within a few weeks. Total IgG levels gradually increased, whereas virus-specific antibodies disappeared after the discontinuation of IgG replacement. The patient was free from life-threatening infectious episodes after allo-SCT.

## Abbreviations

allo-SCT: allogeneic stem cell transplantation
AML: acute myeloid leukemia
ATG: rabbit anti-thymocyte globulin
ATG-F: anti-thymocyte globulin Fresenius
BMT: bone marrow transplantation
BTK: Bruton’s tyrosine kinase
cjKRECs: coding joint KRECs
CsA: cyclosporine
CVID: common variable immunodeficiency
CY: cyclophosphamide
EBV-VCA IgG: EB virus-virus capsid antigen IgG
FLU: fludarabine
G-CSF: granulocyte colony-stimulating factor
GVHD: graft-versus-host disease
GVL: graft-versus-leukemia
Ig: immunoglobulin
IgG: immunoglobulin G
MMF: mycophenolate mofetil
mPSL: methylprednisolone
MTX: methotrexate
PB: peripheral blood
PBSC: peripheral blood stem cell
RAST: radioallergosorbent test
RIC: reduced intensity conditioning
RIST: radioimmunosorbent test
sjKRECs: signal joint kappa-deleting recombination excision circles
STR: short tandem repeat
TBI: total body irradiation
TLI: total lymphoid irradiation
TRECs: T-cell receptor excision circles
XLA: X-linked agammaglobulinemia
